# ‘*I am happy to be listened to’*: co-creation of a simple tool to measure women’s experiences of respectful maternity care in urban Tanzania

**DOI:** 10.1080/16549716.2024.2403972

**Published:** 2024-09-24

**Authors:** Brenda Sequeira D’mello, Natasha Housseine, Hussein Lesio Kidanto, Nanna Maaløe, Jos van Roosmalen, Dan Wolf Meyrowitsch, Thomas van den Akker, Zainab Muniro, Evance Polin, Nuswe Ambokile, Charles Festo, Jane Brandt Sørensen, David Sando

**Affiliations:** a Maternal and Newborn Healthcare, Comprehensive Community Based Rehabilitation in Tanzania (CCBRT), Dar es Salaam, Tanzania; b Medical College, East Africa, Aga Khan University, Dar es Salaam, Tanzania; c Global Health Section, Department of Public Health, University of Copenhagen, Copenhagen, Denmark; d Department of Obstetrics and Gynecology, Copenhagen University Hospital, Herlev, Denmark; e Athena Institute, VU University, Amsterdam, The Netherlands; f Department of Obstetrics and Gynaecology, Leiden University Medical Center, Leiden, The Netherlands; g Health Management Team, Temeke Regional Referral Hospital, Dar es Salaam, Tanzania; h Data Analytics, Ifakara Health Institute, Dar es Salaam, Tanzania; i Management and Development for Health (MDH), Dar es Salaam, Tanzania

**Keywords:** Person-centered maternity care, experience of care, respectful maternity care, measurement tool, disrespect and abuse, mistreatment, urban health, Dar es Salaam

## Abstract

**Background:**

Rights-based Respectful Maternity Care (RMC) is crucial for quality of care and improved birth outcomes, yet RMC measurements are rarely included in facility improvement initiatives. We aimed to (i) co-create a routine RMC measurement tool (RMC-T) for congested maternity units in Dar es Salaam, Tanzania, and (ii) assess the RMC-T’s acceptability among women and healthcare stakeholders.

**Method:**

We employed a participatory approach utilizing multiple mixed methods. This included a scoping review, stakeholder engagement involving postnatal women, healthcare providers, health leadership, and global researchers through interviews, focus groups, and two surveys involving 201 and 838 postnatal women. Cronbach’s alpha and factor analysis were conducted for validation using Stata 15. Theories of social practice and Thematic Framework of Acceptability guided the assessment of stakeholder priorities and tool acceptability.

**Results:**

The multi-phased iterative co-creation process produced the 25-question RMC-T that measures satisfaction, communication, mistreatment (including physical, verbal, and sexual abuse; neglect; discrimination; lack of privacy; unconsented care; post-birth clean-up; informal payments; and denial of care), supportive care (such as food intake and mobility), birth companionship, post-procedure pain relief, bed-sharing, and newborn respect. The pragmatic validation process prioritized stakeholder feedback over strict statistics, lowering Cronbach’s alpha from 0.70 in version 1 to 0.57 for the RMC-T. Women valued the opportunity to share their experiences.

**Conclusions:**

The RMC-T is contextualized, validated, and acceptable for measuring women’s experiences of RMC. Routine use in facility-based quality improvement initiatives, along with targeted actions to address gaps, will advance rights-based RMC. Further validation and community-based studies are needed.

## Paper Context

**• Main findings**: This study describes the participatory approach involving postnatal women, healthcare providers, health leadership, and global researchers to co-create and validate a tool for measuring women’s experiences of respectful maternity care in Dar es Salaam’s urban health facilities.

**• Added knowledge**: The iterative process produced a concise, 25-item Respectful Maternity Care Measurement tool that is user-friendly, administered in 15–20 minutes and addresses all mistreatment domains. The tool reflects women’s priorities and is well accepted by postnatal women and health leaders.

**• Global health impact for policy and action**: Regular use of the tool can enhance awareness of childbirth rights and drive actions to improve and normalize respectful maternity care in low-resource urban settings.

## Background

Persistent challenges in reducing maternal and perinatal mortality and morbidity in resource-constrained countries, despite increasing facility births, reveal critical gaps in the quality of maternity care [1,2]. Respectful Maternity Care (RMC), a crucial yet often overlooked aspect of quality care, is underscored by the World Health Organization (WHO) statement against disrespect and abuse during facility-based childbirth [3]. The statement affirms every woman’s right to the highest attainable health standards, with dignity and respect [3]. Evidence has demonstrated that a lack of RMC violates bioethics, women’s rights, and health standards [4]. Dehumanizing birth experiences, viewed as gender-based violence, undermine trust in the health system, deter facility births, and adversely affect women’s safety and the effectiveness of clinical interventions [5,6]. This is particularly important, as facility births are increasing globally and are promoted as a critical strategy to reduce maternal and perinatal mortality [7].

Over the past two decades, RMC efforts have focused on defining and measuring RMC, understanding its drivers and barriers, and advocating for its integration into routine maternal and newborn care programs [8]. Negative childbirth experiences are described by various terms, with Vogel et al. proposing ‘mistreatment’ as a comprehensive term encompassing physical and verbal abuse, discrimination, detention, extortion, and failures in care, as well as interactions with staff, the facility environment, and the broader health system [9]. This builds on the work of Bowser and Hill, Bohren et al., and Shakibazadeh et al. in defining specific RMC domains [10–12].

In this paper, ‘RMC’ denotes the preferred standard of care, which includes supportive, nurturing care, effective communication, and the absence of mistreatment (care with respect and dignity). When referencing other works, ‘D&A’ and ‘Mistreatment’ will be used interchangeably, adopting the usage employed by the cited author.

Mistreatment of women is a global issue, occurring in both high-income countries (HICs) and low- and middle-income countries (LMICs), though its presentation varies by context [12]. Women in HICs often emphasize shared decision-making and active participation in birth, while women in LMICs seem to prioritize basic care, freedom from verbal and physical abuse, and essential medical care for their and their newborns’ survival and health [12]. Differences between third-party observations and women’s self-reports of mistreatment suggest a normalization of disrespect and abuse in LMICs, highlighting the complex interplay of expectations, accountability, and systemic conditions [13]. This underscores the sensitivity and complexity of RMC and the challenges in developing a single measurement tool that can be universally applicable across different contexts [14].

RMC studies reveal all forms of mistreatment, with a higher prevalence during community follow-up interviews after facility birth [11,15]. Measuring RMC alone cannot eliminate normalized mistreatment, but it is a crucial step towards systematic care improvements [16,17]. Recent efforts have focused on developing RMC measurement tools validated in various countries, including LMICs [18–25]. Tool developers recommend contextual adaptation to enhance usability in local settings [18]. However, routine use of these tools for facility-based quality improvement is rare and few studies document the adaptation process. This represents a missed opportunity to improve maternity care and the broader health system [18].

Tanzania exemplifies a country grappling with an under-resourced health system and low quality of maternal and newborn care [26]. In rapidly urbanizing areas like Dar es Salaam, where nearly all births occur in health facilities, the higher-than-average maternal and perinatal mortality is alarming, with studies showing a higher prevalence of mistreatment in urban areas [27–29]. Consequently, addressing RMC has emerged as a national priority [27–29].

This study builds on a decade-long maternity care quality improvement initiative in 22 public urban health facilities in Dar es Salaam, led by regional leadership and Comprehensive Community-Based Rehabilitation in Tanzania (CCBRT) [30]. The Standards-Based Management and Recognition (SBMR) tool, approved in 2010 by the Tanzanian Ministry of Health, was used to regularly assess the provision of antenatal, intrapartum, and postnatal care within facility-based improvement initiatives. While SBM-R measures some aspects of care experience, such as communication, language, birth position, and privacy, it does not measure mistreatment or practices to avoid, which were identified as gaps by local authors and stakeholders in the CCBRT initiative [30,31].

This study aimed to develop an RMC measurement tool (RMC-T) for routine use in enhancing quality improvement in government health facilities, addressing a gap in current practices. We detail the co-creation, validation, content, and stakeholder acceptability of the RMC-T. ‘Co-creation’ refers to collaborative efforts in participatory research [32].

The co-created RMC-T was integrated into the baseline assessment of the PartoMa scale-up study, which focuses on co-creating and implementing contextual clinical practice guidelines to strengthen childbirth care in five high-volume urban maternity units in Dar es Salaam [33].

## Methods

### Study design

We used a participatory, mixed-methods, human-centered approach based on an evidence-driven adaptive design for health care interventions (AHEAD) to co-create the tool [34]. This empathy-focused approach is useful in generating practical, contextually relevant solutions, placing healthcare providers and pregnant women at the center of the design process. Table 1 details the multi-phased RMC tool co-creation process, which included a literature review, expert reviews, and pilot testing through two surveys in addition to participatory methods [34].

**Table 1.** The phases of the respectful maternity care measurement tool (RMC-T) co-creation process.

Phase	Methodology	People involved, number (n)	Status of tool
**Phase I** Exploration	Direct observations of the infrastructure of study health facilities Literature review about types of disrespect in Tanzania Mapping available tools Selection of tool prototype and RMC domains to be included	Core implementation team (CIRT) n = 5	Zero draft
**Phase II** Stakeholder engagement	Review of the draft tool Online feedback, meetings, and networking In-depth interviews (IDI)	Postnatal women: *n* = 10 Other stakeholders*: n = 12	Draft tool
**Phase III** Pilot Testing	Cross-sectional Survey-1 Validation tests (Component Factor analysis and Cronbach’s alpha test) Analysis of women’s perceptions on the tool and their priorities	Postnatal women in one urban health facility: n = 201	Version 1
**Phase IV** Feedback and Tool revision	Reflection on results from Phase III Focus group discussion (FGD)	Health facility leaders: n = 18 Frontline healthcare workers: n = 10	RMC-T
**Phase V** Acceptability assessment	Cross-sectional Survey-2 Postnatal women’s feedback on the final RMC-T **	Postnatal women in four urban health facilities: n = 838	RMC-T

CIRT: Core Implementation Research Team comprising of co-authors who are researchers and frontline health workers/facility leaders (BSD, NH, ZM, EP, NA, DS).

The CIRT coordinated the co-creation process, integrating feedback into successive tool iterations.

*Other stakeholders: National and international health experts (n = 6) and national professional association of obstetrics and gynecologists (n = 4); public health experts (n = 2).

**Analyzed through the lens of Sekhon’s Total Framework for Acceptability (TFA) [42].

### Study setting

Dar es Salaam, in Tanzania, is one of the world’s fastest-growing cities, with a population approaching seven million [35]. Women from lower-socio-economic backgrounds often opt for subsidized maternity care in the few government-owned public health facilities that offer comprehensive emergency obstetric and neonatal care [36]. The study purposively selected four urban public health facilities from the CCBRT initiative and the PartoMa study. In 2020, these facilities collectively supported 27,450 births [37]. The RMC-T was co-created and validated in one facility, the ‘laboratory health facility’ (Lab-HF), and its final version was assessed for acceptability in three additional facilities. Contextual factors in these facilities, including direct observations of their infrastructure, are summarized in Supplementary File 1. Understanding these urban realities is crucial as they influence the RMC-T’s content and co-creation process.

### Study population

Stakeholders in the co-creation process included postnatal women from the study facilities, front-line workers, midwives, obstetricians, health leaders from the health facilities, district and regional health officials, public health specialists, RMC advocates, and global health researchers. Postnatal women recruited for the surveys (exit interviews) were aged 15 and above and had provided informed consent. Exclusion criteria included women with impaired consciousness during childbirth, post-partum referrals, and those with less than 2 hours of labor before birth in the study hospitals.

### Sample size and sampling procedure for Survey-1 and Survey-2

Sample size calculation for the two surveys was based on an RMC study conducted in a busy urban hospital in Dar es Salaam, which reported a 15% prevalence of disrespect and abuse during childbirth [38]. We used the formula $n=\frac{Z^{2}*P\left(1-P\right)}{E^{2}}$, where *n* = required sample size, Z = 1.96 (at 95% confidence interval), *p* = Percentage of physical abuse, and E = Margin of error around the p-value of 5%. This calculation yielded a minimum sample of 196 participants per health facility. This was rounded up to 200 women from the Lab-HF for Survey-1 and 200 women from each of the four health facilities for Survey-2.

### Data collection

Data collection, spanning from January 2021 to February 2022, employed both qualitative and quantitative methods. The Core Implementation Research Team (CIRT), led by lead and senior authors (BSD/NH and DS) and health facility leadership (NA, EP, ZM), coordinated and steered the co-creation process. Health facility leadership, committed to enhancing RMC, guided the approach, organized IDIs and FGDs, and facilitated research assistants’ (RA) access to postnatal women. DS and BSD have been engaged in RMC advocacy in Tanzania since 2013.

In 2020, CIRT updated a scoping review by Sando et al. on facility-based disrespect and abuse, focusing on physical and verbal abuse from English-language articles in PubMed and Embase [39]. The review used the following search terms: disrespect and abuse, mistreatment, childbirth, disrespect and abuse of childbearing women, mistreatment and women, mistreatment and childbirth [39]. Concurrently, the CIRT mapped international and national tools for assessing mistreatment during childbirth to inform the development of the zero draft for adaptation.

### Qualitative data collection procedures

Participants were briefed on the study objectives and the purpose of the tool. Data were collected on paper by CIRT members for IDIs and FGDs. During in-depth interviews (IDIs) in Phase II, postnatal women reviewed the draft tool and provided feedback on questions’ clarity, relevance, and suggestions for improvement, including tool length and feasibility. During Phase III, three open-ended questions were added to Survey-1: ‘Please mention ways to improve the supportive care/respectful care that you received in this facility,’ ‘During your time in the hospital, would you have liked a birth companion present (e.g. husband, friend, sister, mother-in-law)?’ and ‘Is there anything else you would like to say?’ We included these questions to explore perceptions of birth companionship, a practice not yet implemented in Dar es Salaam at the time of the study. This addition was prioritized by national and global health specialists. Survey-2 (Phase V) included questions to gather perceptions on the tool and interview process: 1) ‘Were the questions understandable?’; 2) ‘How did you feel when answering the questions?’; and 3) ‘How can we improve these questions?’

Supplementary File 2 presents the interview guide used in FGDs with health facility stakeholders, where we assessed the tool’s meaning, clarity, and relevance. Additionally, during this FGD, Survey-1 results were reviewed by facility stakeholders, focusing on tool administration, methods, and potential implementation challenges.

### Quantitative data collection procedures

The CIRT conducted two cross-sectional surveys. Survey-1, from January 2021 to March 2021 (Phase III), piloted version 1 of the tool in the Lab-HF. Survey-2, from December 2021 to February 2022 (Phase V), utilized the final RMC-T.

Survey-1 was conducted by two female research assistants (RAs): a layperson and a social worker. Survey-2 was conducted by five RAs, including the lay person involved for Survey-1, two clinical officers, a medical doctor, and one nurse. They were contracted specifically for this study, and were not part of the health facility staff. All RAs, fluent in Swahili, completed an online course in good clinical practice and a three-day training by BSD, NH, and DS on informed consent, respectful interviewing, and ethical considerations in health research. This training was provided before each survey. The study introduction, or preamble, outlined the study’s purpose and participants’ rights and provided instructions for answering questions based on the current birth experience. RAs were encouraged to follow the preamble precisely and practiced interviewing through role-plays. After completing all interviews, paper-based data were entered into KoBotoolbox.

Each morning, the facility’s director of nursing and midwifery, a CIRT member, briefly introduced the RAs to postnatal women, explained the hospital’s improvement efforts, and invited voluntary participation. The RAs then recruited eligible women, obtained informed consent, and conducted the surveys. In Survey-1, RAs administered all interviews in full. In Survey-2, RAs handled the introduction, consent, eligibility, and socio-demographic information for all participants. For RMC-related questions, RAs interviewed the first 100 women in each facility, while the remaining 100 women self-administered the questionnaire.

### Data analysis

#### Theoretical underpinnings for qualitative data analysis

We used theories of social practice to elaborate on the PartoMa study’s main program theory and explored stakeholders’ perspectives during the tool’s co-creation. We examined how meanings, materials, competencies, motivations, and relations shape care practices and influence behaviors and outcomes [40,41]. Qualitative data were repeatedly reviewed, with inductive coding and deductive thematic analysis, based on social practice theory applied to the co-creation process and open-ended Survey-1 questions [41].

Additionally, we analyzed qualitative data from Survey-2 (Phase V) using Sekhon et al.’s Theoretical Framework of Acceptability (TFA). This framework defines acceptability as ‘a multi-faceted construct that reflects the extent to which people consider a health care intervention to be appropriate, based on anticipated or experienced cognitive and emotional responses’ [42,43]. Data were coded and categorized according to TFA’s seven domains. Sekhon et al. also distinguished between three phases of acceptability: prospective (before participation), concurrent (during participation), and retrospective (after participation) [42,43]. The purpose of this analysis was to understand women’s priorities, guide iterations of the evolving tool, assess validity and acceptability of the RMC-T (Phases II- V), and generate the insights presented in this paper.

#### Quantitative data analysis

Stata version 15 software was used for data analysis. Descriptive statistics were used to analyze and calculate proportions of women’s responses to each RMC-T question. Analysis related to tool validation is presented here, while data on the prevalence of women’s experiences and determinants of RMC are reported elsewhere.

#### Tool validation

We aimed to optimize the RMC-T for content, construct, understandability, and ease of use for frontline managers to measure RMC and integrate findings into quality improvement initiatives. A pragmatic validation approach was selected to ensure its effectiveness.

Content validity was assessed to determine if the RMC-T measured women’s experiences of respectful care and to identify items for inclusion [34,44]. This was achieved through a literature review and feedback from midwives, obstetricians, public health specialists, and RMC advocates, provided via discussion or via email [34]. The factor structure of the questions was evaluated through exploratory factor analysis for each component of version 1 of the tool [44]. Kaiser–Meyer–Olkin (KMO) and Bartlett’s test of sphericity assessed the data suitability for factor analysis [44]. Internal consistency reliability was determined using Cronbach’s alpha, with a value of ≥0.70 indicating a good reliability [44].

#### Ethical approval

The study received ethical approval from the National Health Research Ethics Review Committee (NatHREC) at the National Institute for Medical Research (NIMR/HQ/R.8c/Vol. I/2539) and a permission letter from the Ministry of Health, Tanzania. Written consent for exit interviews was obtained from the heads of the participating hospitals (one facility refused consent, as detailed in the results). Informed consent was obtained from all women and participants in IDIs and FGDs. A standardized Swahili preamble was read aloud to explain the study’s purpose, data collection procedures, participants’ rights, and the voluntary nature of participation, with verbal consent recorded. The authors acknowledge the power dynamics involved in discussing women’s birth experiences, especially negative ones, within a health facility setting. This issue was addressed in the RAs’ training, which emphasized the importance of empathy and sensitivity during participant interactions. Additionally, the preamble highlighted the tool’s purpose for quality improvement.

## Results

The results are presented according to the five phases of the RMC-T co-creation process: exploration, stakeholder feedback, Survey-1 (pilot testing), validation, tool refinement process, and acceptability testing, as outlined in Table 1.

### PHASE I: exploration and initial tool development

This phase involved observing hospital infrastructure, materials, personnel, and policies affecting care practices (Supplementary File 1); the scoping review on physical and verbal abuse (Supplementary File 3); and the international and national RMC measurement tools were reviewed by CIRT in 2020 to inform the zero draft (Supplementary File 4) [18,20,38,45].

The zero draft was based on a comprehensive community survey tool developed by Bohren et al. in collaboration with the WHO quality of care group. This tool, which was grounded on a global review of 65 studies across 34 countries, was selected for its thorough coverage of common mistreatment types relevant to Tanzania, inclusion of all RMC domains and alignment with the RMC charter [11,45,46]. The CIRT extensively and iteratively revised this draft, tailoring it to retain mistreatment types specific to Tanzania while streamlining it for practical routine use. Modifications included removing questions on mistreatment timing, perpetrators, future childbearing, and postpartum depression; merging similar items; and adopting a yes/no/I don’t know format, preferred for its simplicity over the Likert scale (which was considered lengthy and difficult to understand during Swahili translation). By the end of Phase I, the zero draft of the tool included 57 RMC-related questions, incorporating item types and phrasing from existing tools and organized into relevant RMC sections.

### PHASE II: stakeholder review

National and international public health specialists provided feedback on the zero-draft tool via email. Comments mainly focused on word choices, reordering questions to start with less sensitive ones, integrating questions on newborn care practices, and suggestions to include a question on birth companionship.

Postnatal women recommended improving Swahili terms by replacing technical and academic Swahili terms with commonly used language. For example, the term for birth companionship (‘*Mwambata*’), derived from previous RMC research conducted in Tanzania, was replaced by a more explanatory term [47]. The revised question regarding birth companionship now reads: ‘*Je uliruhusiwa kuwa na mtu wako wa karibu wakati unajifungua? Mfano: - mama, rafiki, dada, mama mkwe au mwenza wako?’* (In English: Were you allowed to have a person close to you during birth, for example, your mother, friend, elder sister, mother-in-law or your partner?)

Postnatal women expressed willingness to participate in the interviews, confirmed understanding of the questions, and preferred interview times between 10 am and 3 pm, after morning rounds and before the time of discharge and visiting hours. Surprisingly, women preferred answering questions on their ward beds, near their babies and belongings, where they could observe ward activities, rather than in a private side room that was made available for the interviews. This context of time, place, and space was crucial for their participation. Additionally, some of their inquiries about the study’s purpose prompted improvements to the standardized written preamble used for introducing the study and obtaining consent. Incorporating women’s feedback resulted in a 49-item questionnaire (version 1 of the tool used for Survey-1).

### PHASE III: pilot testing of the tool (version 1)

Between January and February 2021, the first survey was conducted with 201 consecutively recruited postnatal women in the Lab-HF. Table 2 presents their sociodemographic characteristics, while Table 3 shows the findings from Survey-1 conducted using version 1 of the tool. The synthesis of women’s perceptions of care and the tool are summarized below, including key findings and sample responses.

**Table 2.** Sociodemographic characteristics of 201 postnatal women in Survey-1.

Parameter	Vaginal birth (n = 114)	Caesarean section (n = 87)	Total (n = 201)
Age (in years) [Median, (IQR)]	26 (22-31)	26 (22-31)	26 (22-31)
**Marital status**			
Single	14 (12.3)	9 (10.3)	23 (11.4)
Married/cohabiting	100 (87.8)	78 (89.6)	178 (88.5)
**Level of education**			
No education/Incomplete primary education	26 (22.8)	13 (14.9)	39 (19.4)
Complete primary education	31 (27.2)	28 (32.2)	59 (29.4)
Complete secondary (Form1V)	43 (37.7)	37 (42.5)	80 (39.8)
Any post-secondary education	14 (12.3)	9 (10.3)	23 (11.4)
**Parity**			
Primiparous	46 (40.4)	38 (43.7)	84 (41.8)
Multiparous	68 (59.6)	49 (56.3)	117 (58.2)

Values are presented as number (percentages) unless otherwise specified.

**Table 3.** Prevalence of RMC in Survey-1 and subsequent tool adjustments.

Variable (type of mistreatment)	Number of women who responded affirmatively N (%)	Adjustments (Phase IV)
**A. Verbal abuse**		
1. Shout/scream/scold you	19 (9.5)	Retained Merged into one Verbal abuse question with explanatory remarks. During Swahili translation the questions seemed similar.
2. Insult or mock you	9 (4.5)	
3. Use bad/abusive language	9 (4.5)	
4. Make negative comments about your physical appearance (such as your weight, private parts, cleanliness, or other parts of your body)	3 (1.5)	
5. Make negative comments about your baby’s physical appearance (such as his/her appearance, sex, or other aspects of the baby)?	3 (1.5)	
6. Make negative comments to you regarding your sexual activity?	3 (1.5)	
7. Threaten you with a medical procedure (such as episiotomy or caesarean section)	4 (2.0)	
8. Threaten you with physical violence?	3 (1.5)	
9. Frighten you that something bad would happen if you didn’t cooperate well.	13 (6.5)	
10. Threaten to withhold care from you or your baby	7 (3.5)	
11. Blame you for something that happened to you or your baby during your time in hospital	5 (2.5)	
**B. Physical abuse**		
1. Kick/slap/punch or hit with something	3 (1.5)	Retained Merged into physical abuse question with explanatory remarks
2. Gag/physically restrain (e.g. being tied or held to the bed)	1 (0.5)	
3. Apply forceful downward pressure on your abdomen before the baby came out (fundal pressure)	4 (2.0)	
4. Any sexual abuse, or inappropriate sexual language or touch?	0	
5. You had another form of physical force used against you.	2 (1.0)	
**C. Stigma and Discrimination (any)**		
1. Your ethnicity, race, tribe, culture or religion, your skin color, or the color of your baby?	3 (1.5)	Retained Merging into stigma and discrimination question as explanatory remarks
2. Your age?	4 (2.0)	
3. Whether you were married or not?	1 (0.5)	
4. Your level of education or literacy?	3 (1.5)	
5. Your economic circumstances?	5 (2.5)	
6. Your HIV status?	2 (1.0)	
7. Your physical status – if any disability	1 (0.5)	
**D. Non-private care during childbirth (any)**		
1. Curtains, partitions, or other measures were NOT used to provide privacy for you from other patients, patients’ family members, or health workers/staff not involved in providing you care	23 (11.4)	Retained Reworded into two separate questions on physical privacy and informational privacy
2. Not all vaginal and physical examinations conducted privately (in a way that other people could not see)	19 (9.5)	
3. Shared a bed/or mattress with another woman or women	125 (62.2)	Upgraded into a stand-alone question
**E. Supportive care-mobilization, fluids, and pain relief**		
1. No access to water to drink	14 (7.0)	Retained but merged
2. Not allowed to eat	5 (2.5)	
3. Not told that you could walk or move around during labor	82 (40.8)	Retained
4. No pain relief (e.g. comforting, pain medication)	111 (55.2)	Retained and rephrased to be more specific to pain relief after operative procedures
**F. Unreasonable Demands, Fee Structures**		
1. Immediately after birth, were you instructed to clean up your own blood, urine, feces, or birth-fluid from the birthing area/bed?	14 (7.0)	Retained as a separate question
2. Did staff suggest or ask you (or your family or friends) for a bribe, informal payment, or gift?	7 (3.5)	Retained and rephrased as a separate question
3. Where you or your baby denied treatment due to inability to pay?	8 (4.0)	Retained
4. Were you or your baby detained in the hospital/not allowed to be discharged due to inability to pay hospital bills?	128 (63.7)	Deleted Unclear question within policy of free maternity care and similar to bribe
**G. Non-consented Care**		
1. Did you receive any procedure without being given any explanation beforehand? (e.g. vaginal examination, caesarean section, episiotomy, hysterectomy, tubal ligation/sterilization, post-partum IUCD, manual removal of placenta, assisted vaginal delivery)	6 (5.3)	Deleted. Similar to consented procedure
2. Did you receive any procedure without your consent/or you agreeing to the procedure (e.g. vaginal examination, caesarean section, episiotomy, hysterectomy, tubal ligation/sterilization, post- partum IUCD, manual removal of placenta, assisted vaginal delivery, induction)	6 (5.3)	Retained and rephrased to improve clarity
**H. Disrespect to the newborn baby**		
1. Baby separated from you without information/explanation?	4 (2.0)	Retained
2. Did not receive information about the condition of your baby.	58 (28.9)	Retained
3. Procedure performed on your baby without informing you.	1 (0.5)	Deleted as the questions were unclear
4. Did not know if procedure performed on baby.	29 (14.4)	
**I. Emotional Support during labor and childbirth**		
1. Did not feel you were received well/welcomed on admission to the hospital	12 (6.0)	Retained and rephrased by merging with I-5 (encouraging kind words)
2. Feel ignored/neglected by the health workers or staff	22 (10.9)	Retained
3. Wait for long periods of time before you were attended by health workers	48 (23.9)	Removed as in context was similar to neglect
4. No health worker present when the baby came out?	6 (3.0)	Removed as in context was similar to neglect
5. Health workers did not say kind or encouraging words to you?	35 (17.4)	Retained by merging in one question with I-1
6. No opportunity to discuss any concerns you had with a health worker/ask questions/share decision-making on preferences/requests?	132 (65.7)	Retained and rephrased
7. Where you satisfied with the care you received during your stay at the hospital for childbirth?	181 (90.0)	Removed
8. Would recommend this facility to other women for birth?	178 (88.6)	Retained
**J. Women’s suggestions for improving childbirth care in this facility (open-ended questions for discussion)** Please mention ways to improve the supportive care/respectful care that you received at this facility During your time in hospital, would you have liked to have a birth companion present? For example, your husband, a friend, sister, mother-in-law, etc.? Is there anything else you would like to say? Do you have any questions for me?		Qualitative analysis resulted in additional questions*
*Questions that were added during Phase IV *Do you think there were enough health staff in the facility to care for you?* *Do you think the facility had a clean environment and good toilet and washing facilities?* *Do you think there were enough medicines and supplies in the facility to care for you?* *Were you allowed to have someone you know, a close family member to stay with you during childbirth such as your mother, a friend, sister, mother-in-law, or your partner?*		

### Women’s understanding of the questions in the tool

Women’s responses suggested that they understood the questions. They demonstrated a willingness to answer all questions and supplemented their responses with comments expressing satisfaction and pleasure in participating and being offered an opportunity to share their birth experiences.

**Table:** 

Sample responses suggesting understanding *‘The questions were not that hard, and I am happy to be listened to …my opinions will help improve care for mothers’* *‘I have no questions. I liked the questions and also got an opportunity to air out my opinions’* ‘*I have no questions; I feel great taking part in the study because as mothers we face challenges and struggles, and we need to be listened to so that the challenges and struggles are worked upon because we have a right to be listened to’* *(postnatal women, open-ended response – survey 1)*

### Socio-cultural differences concerning understanding of pain relief and childbirth rights

Findings from FGDs with healthcare providers and leaders revealed cultural differences in understanding labor pain and pain relief. The question about pain relief did not sufficiently distinguish between labor pain and post-surgical pain. In a setting where obstetric analgesia is reserved for cesarean sections, we were advised to focus on pain experienced during and after cesarean sections, episiotomies, and perineal repairs. Additionally, beliefs influence perceptions and management of labor pain, with some tribes expecting women to endure pain ‘bravely and quietly.’ Participants noted that women who cried loudly were from tribes that did not have ‘pain-bearing’ training for birthing women.

Importantly, during interviews, some women asked, ‘Is this how it is supposed to be?’ indicating a lack of awareness about childbirth rights.

### Self-administration and reduction in length of the tool

Requests to shorten the tool were voiced by postnatal women, frontline healthcare workers, and hospital leadership. In FGDs, hospital leaders noted similarities in some questions when translated into Swahili. These considerations led to reducing sub-questions, reformatting for self-administration, and allowing data collection to be managed by a single assistant. Discussions concluded that, despite staff shortages limiting capacity, the quality and social care department should be responsible for tool administration and managing mistreatment complaints.

### Women’s perceptions about care experienced and women’s priority request ‘not to share beds’

Some women expressed only positive perceptions of the care they received, for example, some postnatal women interviewed in Survey-1 mentioned feeling that ‘*everything is ok, there is no need to change*’. However, most responses expressed a coexistence of positive and negative experiences. The most common complaint involved material gaps, like bed-sharing and the lack of mosquito nets. In Survey-1, over half of the women (125, 62.2%) reported having to share beds.

**Table:** 

Examples of responses suggesting material gaps *‘They should improve availability of more beds, but the care is great; I told them that I was scared, and they took care of me until I got my baby’* *‘I shared a bed since labor until postnatal so they should increase the number of beds and mosquito nets you cannot sleep at night because you are trying to make sure that the baby doesn’t get mosquito bites, but other care was ok’* *(postnatal women, open-ended response – survey 1)*

Additional complaints related to substandard care practices, for example, lack of privacy, inadequate use of pain relief after caesarean section, and unconsented care.

**Table:** 

Examples of responses suggesting sub-standard care practices ‘*Childbirth care should be done with privacy. When I was giving birth, everyone passing by would come to see me. There was a curtain, but when someone arrived, they would pull it aside*.’ ‘ *… There should be more improvement in availability of the medicine around because since I left the theatre, I have been asking for painkillers but was told to wait until the doctor comes.’* *‘…do not administer labor-inducing drugs to a woman without her consent.’* *(postnatal women, open-ended response – survey 1)*

Finally, women emphasized *social relations*, particularly the importance of establishing a kind rapport between themselves and healthcare providers.

**Table:** 

Examples of responses revealing postnatal women’s demand for kind comforting language ‘*Let health workers not be critical but instead, have love and offer words of comfort to women. May there be consistent (continuous support during labor) and may the government help us*.’ ‘ *… birth attendants should listen to women and not scold or embarrass them.’* *(postnatal women, open-ended response – survey 1)*

**Table:** Box 1: Respectful maternity care measurement tool.

Questions in the Respectful Maternity Care Tool	Please indicate with a √ mark your answer if it is yes or if it is no, or I don’t know for the following questions. If there is an explanation, write it in the margin.
1.	Did the healthcare workers treat you kindly by for example speaking to you in a kind voice, smiling at you, making you feel welcomed and saying encouraging and comforting words to you during your pain?
2.	Do you feel like the healthcare workers at the facility gave you a chance to ask questions and listened to your concerns and wishes?
3.	Did the healthcare workers explain to you why they were doing examinations or procedures on you or giving you medication?
4.	During your time in hospital did you receive any medication or procedures without permission/consent beforehand? Examples of procedures include vaginal examination, caesarean section, episiotomy, removal of the uterus, tubal ligation, post- partum IUCD, manual removal of placenta, assisted vaginal delivery.
5.	Do you feel healthcare workers neglected or ignored you?
6.	Do you feel you received poor care because of any of the following: your physical appearance, ethnicity, race, tribe, culture or religion, age, marital status, number of children you have, your education, wealth, inability to pay hospital bills, HIV status?
7.	Were you allowed to have someone you know, a close family member to stay with you during childbirth such as your mother, a friend, sister, mother-in-law, or your partner?
8.	Were you told that you could walk and encouraged to move around during labor?
9.	Were you allowed to eat and drink when you wanted to or when you were thirsty/hungry?
10.	Was pain medication given during and after painful procedures, for example during repair of the perineum and after caesarean section?
11.	Did a healthcare worker talk to you rudely? For example, did anyone shout at you, scold you, insult you, frighten you that something bad would happen if you didn’t cooperate or blame you for something that happened to you or your baby during your time in hospital?
12.	During your stay in the hospital or during childbirth did any staff or anyone physically hurt you for example by doing any of the following: kicking, slapping, pushing, hitting, pressing hard on your abdomen, pinching you with fingers or any sharp instrument or cover your mouth or physically tie you up?
13.	Did any healthcare worker make a flirting comment, or use inappropriate sexual language or requested you to have sex with them?
14.	Do you feel that the care providers talked to you about your information out loud or in a way that other mothers or other health workers not involved in your care could hear?
15.	During examinations in the ward, were you always covered up with a cloth or curtain so that you did not feel exposed?
16.	At any time, did you have to share a bed/or mattress with another woman or women?
17.	Immediately after birth, were you instructed to clean up your own blood, urine, feces or birth fluid from the birthing area/bed?
18.	Did healthcare providers suggest or ask you (or your family or friends) for a bribe, informal payment, or gift?
19.	Did you receive information about the condition of your baby during labor and after birth?
20.	Was your baby separated from you after birth without information/explanation?
21.	Were you or your baby denied treatment due to inability to pay?
22.	Do you think there was enough health staff in the facility to care for you?
23.	Do you think the facility had a clean environment and good toilet and washing facilities?
24.	Do you think there were enough medicines and supplies in the facility to care for you?
25.	Would you recommend this facility to other women for giving birth?

Note: For clarity, BOX 1 includes only the 25 RMC-specific questions. The introductory section, consent information, response sections (‘Yes,’ ‘No,’ ‘Don’t Know,’ ‘Explain’), and socio-demographic details are not included. The full English and Swahili versions of the questionnaire are provided as Supplementary files 7 and 8.

### Validation test results summary

The validation analysis performed on data from Survey-1 (Table 4) showed six domains had Cronbach’s alpha above 0.70, including verbal abuse, physical abuse, stigma and discrimination, consented care, emotional support, and privacy. Three domains had Cronbach’s alpha below 0.70: privacy, mobilization, fluids and pain relief, unreasonable demands, and newborn (dis)respect.

**Table 4.** Summary results of the validation analysis of version 1 of the tool.

Domain name	Items	Cronbach Alpha (CA) (range of CA for item deletion)	Correlation	Exploratory Factor Analysis (EFA)	Reflection
Verbal Abuse	11	0.9116 (0.8988 to 0.9211	Between 0.5484 and 0.8722	Two factors loading Three items did not load	Retain and merge. Delete the three non-loading items*
Physical Abuse	4	0.7115 (0.4479 to 7471)	Between 0.5862 and 0.9048	One factor loading	Retain
Stigma and Discrimination	7	0.9429 (0.9220 to 0.9620)	Between 0.3960 and 0.9370	One factor loading	Retain
Privacy	3	0.4417 (0.0709 to 0.9450)	Between 0.0152 and 0.5304	One factor loading	Review and revise
Mobilization, fluids/pain relief	4	0.2232 (0.1081 to 0.2344)	Between 0.0542 and 0.2016	One factor loading	Review and revise
Unreasonable demands	4	0.2845 (0.0376 to 0.3421)	Between 0.0279 and 0.2613	One factor loading with three items	Review and revise
Consented care	2	0.9060	NA	One factor loading	Retain
Newborn (dis) respect	3	0.0077 (0.0077 to 0.0253)	Between 0.077 and 0.0456	One factor loading	Review and revise
Emotional support	8	0.7058 (0.6114 to 0.7507)	Between 0.0022 and 0.7407	Two factors loading	Retain and merge

Sample size = 201. Scale description: yes/no variable scale coded as 1 = yes, 0 = No, 2 = Don’t know (excluded); Don’t know responses per domain (number): F (19); H (29); I (2); for all other domains, all 201 responded.

*Items not loading were related to negative comments about the baby and threats of physical violence, which in Swahili were similar to terms for physical or verbal abuse.

### PHASE IV: tool refinement (based on reflection and feedback from Phase III)

Feedback from postnatal women, healthcare providers, facility leadership, and statistical tests guided the iterations of version 1. Tables 3 and Table 4 indicate the rationale for some changes from version 1 to the final RMC-T. For example, sub-questions related to items like physical and verbal abuse were consolidated into explanatory remarks. New questions addressed women’s priorities, such as bed sharing, clean environment, staffing, medicines, and equipment. Systematic reordering, recommended by international stakeholders, resulted in placing less intrusive questions first. Subtle adjustments to language, font, style, and the preamble enhanced self-administration. The pragmatic decision to retain all relevant items, reduce sub-scales, and include stand-alone questions reduced the number of questions from 46 to 25, finalizing Phase IV and the co-creation process. Consequently, Cronbach’s alpha decreased to 0.5699 for the final 25-item RMC-T (Supplementary File 5).

### The final RMC tool

The finalized RMC-T features 25 items (Box 1), with ‘yes/no/explain’ options, suitable for assisted or self-administration, and can be completed in 15–20 minutes. The tool is available in Swahili and English (Supplementary Files 7 and 8). The questions are categorized into themes that align with the rights of childbearing women and the WHO quality of care standards: effective communication (three questions); dignity/respect (includes inquiry for 11 types of mistreatment including sexual abuse); supportive care (three questions); infrastructure/material constraints (four questions); newborn disrespect (two questions), a single question each on pain relief (related to procedures) and satisfaction with care [4,48]. Final adjustments were guided by women’s priorities, clinical relevance, and the tool’s focus on identifying actionable gaps in RMC. Supplementary Table 6 provides a basic comparison of RMC-T items with those in similar tools [18,20,46,49].

### PHASE V: acceptability of the RMC-T

Table 5 presents the RMC-T’s acceptability based on qualitative feedback from open-ended questions in Survey-2, conducted from 11 December 2021 to 24 February 2022. This survey included 838 postnatal women from four urban health facilities: 402 were interviewed by RAs, and 436 completed self-administered questionnaires. Details on the prevalence of RMC are provided in a separate, forthcoming manuscript.

**Table 5.** RMC-T acceptability evaluated using the theoretical framework of acceptability (TFA)*.

Acceptability Constructs	Sample responses
*Affective attitude* How an individual feels about taking part in an intervention	‘*It feels great to be asked these questions, it feels like you care.*’ (Postnatal woman – Survey-2) ‘*Interviews like this should be ongoing.*’ (Postnatal woman – Survey-2)
*Burden* The amount of effort required to participate in the intervention.	Interviews took about 15 minutes – (observation) ‘*The questions are many, but very important.*’ (Postnatal woman – Survey-2) *Five women refused to participate in Survey-1 (reasons included the baby crying or being restless or if the postnatal woman was experiencing after-birth abdominal pains)*. RAs noted that women would be restless and more likely to answer, ‘I don’t know’ when the baby started crying.
*Ethicality* The extent to which the intervention has good fit with an individual’s value system.	‘*I would like to see my concerns worked on.*’ ‘*I feel great to participate because there are challenges that mothers face and we don’t know where or whom to report to but with such studies it helps.*’ (Postnatal woman – Survey-2)
*Perceived effectiveness* The extent to which the intervention is perceived as likely to achieve its purpose.	This was not assessed.
*Intervention coherence* Refers to the extent to which participants understood how the intervention works and its importance.	In Survey-2, 68.5% of the 838 women provided feedback indicating they understood the questions. ‘*I understood the questions*.’ ‘*These are true questions*.’ ‘*No need to change or improve the questions*.’ ‘*I loved them, I feel great*.’ One woman recommended strengthening the preamble for self-administered questionnaire, she said: ‘*I understood the questions, but you can improve the explanation for more clarification.’* The quality and safety officer found the tool’s questions relevant and aligned with verbal complaints. Health leaders valued the tool for its ability to proactively detect mistreatment and address issues before they escalated into political, media controversies, or major patient complaints.
*Self-efficacy* The participant’s confidence that they can perform the behaviors required to participate in the intervention.	436 women self-administered the questionnaire. Overall, ‘don’t know’ responses were low (1.3%), but slightly higher in the self-administered group (2.5%) compared to the research administered group (0.1%). Health leaders found the yes/no questions straightforward to use and analyze, noting their similarity to familiar quality tools.
*Opportunity costs* The extent to which benefits, profits or values must be given up engaging in an intervention.	One of the five identified health facilities declined participation, citing concerns that RMC-T questions on bed-sharing and birth companions might reflect poorly on their facility due to high workload and staff shortages.

*Sekhon et al. [43].

## Discussion

The final 25-item RMC-T, developed through extensive input from postnatal women, healthcare providers, and public health experts, comprehensively covers all RMC domains and aligns with women’s priorities. Designed for completion in 15–20 minutes, the tool is user-friendly for frontline managers, targeting specific mistreatments to enable actionable improvements. Both women and healthcare providers found the questions relevant and reflective of their realities.

Version 1 of the tool demonstrated high internal consistency with Cronbach’s alpha exceeding 0.70. The pragmatic co-creation process prioritized local stakeholder feedback over strict statistical validation resulting in lowering Cronbach’s alpha to 0.57 for the RMC-T. Rigorous validation can sometimes omit important items, a methodological trade-off acknowledged by developers [18,20]. For example, Sheferaw et al. noted that despite identifying experiences of consented and confidential care, exploratory factor analysis could not extract these components, leading to their exclusion [20]. Similarly, Afulani et al. excluded discrimination and privacy due to ‘low factor loading’ but noted that ‘even though these items did not make it to their current scale, they are important to consider as stand-alone questions in RMC research as they may be important in other settings’ [18].

With these considerations, RMC-T retained contextually relevant items from all RMC domains, even those with low validation test scores such as sexual abuse, privacy, unstructured fee payments, pain relief, and newborn respect [38,50,51]. It excluded elements like local language use and choice of birth position covered by the SBM-R tool and omitted aspects like trust and feelings of safety, which are challenging to define in settings with economically disadvantaged women who may have low care expectations [52]. The final RMC-T aligns well with other RMC tools, as noted in Supplementary Table 6.

Sharing hospital beds emerged as a major concern for postnatal women. Bed sharing is surprisingly prevalent not only in urban areas but also in rural areas, as shown by studies in Tanzania and Ethiopia [14,28,53]. Bed-sharing, though documented in qualitative studies, is often excluded from RMC tools [18,20,24,54]. This may be due to its normalization or the adaptation of tools from high-income countries where hospital bed-sharing is inconceivable. The omission overlooks the risks of bed-sharing, including compromised privacy, infection (especially during COVID-19), and its association with overcrowding that undermines clinical care and the overall care experience. Incorporating bed-sharing into the RMC-T could highlight the problem and facilitate advocacy for context-specific reforms, as suggested by Freedman et al. [55].

A key difference in the RMC-T is the use of a simple ‘yes/no/explain’ response option instead of a Likert scale. This choice was made for ease of administration and translation challenges in low-resource settings [52]. While Likert scales provide greater precision, Scott et al. found them ‘generally incomprehensible’ to pregnant and postpartum women in rural India, who were unfamiliar with graduated levels of agreement, particularly among less educated populations [52]. This sentiment was echoed by local stakeholders and may apply to other low-resource settings.

### Strengths and limitations

The major strength of this study lies in its participatory co-creation approach, which integrates human experiences and local contexts. It shifts from traditional top-down knowledge translation to a more collaborative model. It draws on extensive local and international expertise and builds upon prior RMC studies [38,56]. The high acceptability of the tool reflects the effectiveness of this participatory approach. Social practice theories guided the integration of women’s priorities into the tool development [41]. Sekhon et al.’s Theoretical Framework of Acceptability provided a comprehensive approach for evaluating ‘prospective acceptability’ [42,43]. Despite concerns about exit interviews and lower mistreatment reports, women expressed that they valued discussing their birth experiences during their hospital stay.

This study, however, has several limitations. Under-reporting is a concern due to factors such as the timing (exit interviews), location of interviews (postnatal ward), courtesy bias, societal norms, low expectations of care, fear of disclosure, and normalization of mistreatment. These factors should be considered when interpreting results. The tool identifies RMC gaps rather than providing precise measurements [13]. While supplemental and repeated community-based interval interviews are recommended for a deeper understanding of the issues, they are not feasible for routine and regular measurements required for quality improvement.

Selection bias during exit interviews presents a further limitation. This approach was chosen for its feasibility and alignment with the study’s purpose to enable facility-based quality improvement, identify mistreatment close to the time of the incident, amplify women’s voices, and minimize additional health system costs.

While ‘don’t know’ responses are common in surveys, the 2.5% ‘don’t know’ responses in the self-administered group compared to 0.1% in the research-administered group indicate a need for improved self-administration processes. Enhancing education on women’s rights during birth could also improve tool performance.

The tool was tailored for high-volume urban public health facilities and integrated into the CCBRT and PartoMa Project, which limits its generalizability to smaller or private facilities [30,33]. While its performance in these settings is uncertain, the co-creation process can be adapted to various contexts. For instance, the PartoMa project, initially implemented in Zanzibar, has been adapted through co-creation for urban and rural hospitals in Dar es Salaam and Ethiopia [33]. Further research is needed in rural settings and post-discharge interviews.

Additionally, the RMC-T does not capture healthcare providers’ well-being, which is crucial for RMC; addressing this may require a separate tool.

Studies suggest that international RMC concepts, such as satisfaction, may not resonate universally due to varying worldviews and contextual realities, strong adherence to medical authority, limited awareness of alternatives, entrenched gender norms, low expectations of care, and normalization of poor care [52]. This raises concerns about applying global constructs across diverse populations and highlights the importance of ‘context’ and the ongoing challenge of establishing gold standard indicators for social constructs such as satisfaction and respectful care [57]. This underscores the need for further local research, ideally conducted along with community education on universal health rights.

### Clinical implications

The RMC-T can be integrated into routine maternal health quality improvement efforts to address mistreatment and better meet women’s needs [58]. Used in the PartoMa study baseline assessment in Dar es Salaam, the tool effectively bridged the gap between tool developers and users [33]. The tool can be administered with minimal training, allowing various cadres, including laypersons, to deploy it effectively. Offering both self-administration and assisted options for a randomly selected subset of birthing women is suggested as a solution to human resource challenges, to enhance inclusivity for those with lower literacy and provide valuable insights into RMC gaps. Facility leadership, with support from the district leaders and women beneficiaries, can determine the most appropriate option for their context. Educating women on childbirth rights and presenting regular RMC measurements during quality improvement meetings can raise awareness of rights-based respectful care, improve tool performance, drive real-time actions to reduce urban health disparities, and promote RMC integration across Tanzania.

## Conclusion

RMC is a complex issue for women in urban, low-resource settings during facility-based childbirth. The 25-item RMC-T is a user-friendly tool that effectively identifies areas for improving RMC and is well received by both postnatal women and health facility leadership. Regular use of the tool, combined with targeted actions to address gaps, will advance RMC fostering rights-based respectful care. Further innovations in community administration and validation studies across diverse settings are needed, along with efforts to raise awareness about women’s rights during childbirth. As global advocacy for facility births grows, this tool may also benefit rural areas facing similar RMC challenges.

## Supplementary Material

SupplementaryFile8_Tool_ENGLISH.docx

SupplementaryFiles1_6.docx

## References

[1] Sharrow D, Hug L, You D, et al. Global, regional, and national trends in under-5 mortality between 1990 and 2019 with scenario-based projections until 2030: a systematic analysis by the UN inter-agency group for child mortality estimation. Lancet Glob Health. 2022;10:e195–15. doi: 10.1016/S2214-109X(21)00515-5 35063111 PMC8789561

[2] Kruk ME, Gage AD, Joseph NT, et al. Mortality due to low-quality health systems in the universal health coverage era: a systematic analysis of amenable deaths in 137 countries. The Lancet. 2018;392:2203–2212. doi: 10.1016/S0140-6736(18)31668-4 PMC6238021 30195398

[3] World Health Organization. The prevention and elimination of disrespect and abuse during facility-based childbirth. Geneva: World Health Organization; 2014.

[4] World Health Organization. WHO recommendations: intrapartum care for a positive childbirth experience. Geneva: World Health Organization; 2018. 30070803

[5] Downe S, Finlayson K, Oladapo O, Bonet M. Correction: what matters to women during childbirth: a systematic qualitative review. PLOS ONE. 2018;13:1–17. doi: 10.1371/journal.pone.0197791 PMC5903648 29664907

[6] Doyle C, Lennox L, Bell D. A systematic review of evidence on the links between patient experience and clinical safety and effectiveness. BMJ Open. 2013;3:3. doi: 10.1136/bmjopen-2012-001570 PMC3549241 23293244

[7] Kruk ME, Gage AD, Arsenault C, et al. High-quality health systems in the sustainable development goals era: time for a revolution. The Lancet Global Health Commission High Qual Health Syst SDG Era Lancet Global Health. 2018:e1196–252. 10.1016/S2214-109X(18)30386-3 PMC7734391 30196093

[8] Bohren MA, Hunter EC, Munthe-Kaas HM, et al. Facilitators and barriers to facility-based delivery in low- and middle-income countries: a qualitative evidence synthesis. Reprod Health. 2014;11:1–17. doi: 10.1186/1742-4755-11-71 25238684 PMC4247708

[9] Vogel JP, Bohren MA, TuncßalpӦ OO, et al. Promoting respect and preventing mistreatment during childbirth. BJOG. 2016;123:671–674. doi: 10.1111/1471-0528.13750 26628382 PMC5063112

[10] Bowser D, Hill K. Exploring evidence for disrespect and abuse in facility-based childbirth; report of a landscape analysis. (WA) (DC); 2010.

[11] Bohren MA, Vogel JP, Hunter EC, et al. The mistreatment of women during childbirth in health facilities globally: a mixed-methods systematic review. PLOS Med. 2015;12:1–32. doi: 10.1371/journal.pmed.1001847 PMC4488322 26126110

[12] Shakibazadeh E, Namadian M, Bohren MA, et al. Respectful care during childbirth in health facilities globally: a qualitative evidence synthesis. BJOG. 2018;125:932–942. doi: 10.1111/1471-0528.15015 29117644 PMC6033006

[13] Freedman LP, Kujawski SA, Mbuyita S, et al. Eye of the beholder? Observation versus self-report in the measurement of disrespect and abuse during facility-based childbirth. Reprod Health Matters. 2018;26:107–122. doi: 10.1080/09688080.2018.1502024 30199353

[14] Asefa A, McPake B, Langer A, et al. Imagining maternity care as a complex adaptive system: understanding health system constraints to the promotion of respectful maternity care. Sex Reprod Health Matters. 2020;28:1. doi: 10.1080/26410397.2020.1854153 PMC7888043 33308051

[15] Kruk ME, Kujawski S, Mbaruku G, et al. Disrespectful and abusive treatment during facility delivery in Tanzania: a facility and community survey. Health Policy Plan. 2018;33:e26–33. doi: 10.1093/heapol/czu079 29304252

[16] Agweyu A, Hill K, Diaz T, et al. Regular measurement is essential but insufficient to improve quality of healthcare. BMJ. 2023;380–e073412. doi: 10.1136/bmj-2022-073412 36914202 PMC9999465

[17] Marx M, Nitschke C, Nafula M, et al. If you can’t measure it- you can’t change it – a longitudinal study on improving quality of care in hospitals and health centers in rural Kenya. BMC Health Serv Res. 2018;18:1. doi: 10.1186/s12913-018-3052-7 29622012 PMC5887241

[18] Afulani PA, Diamond-Smith N, Golub G, et al. Development of a tool to measure person-centered maternity care in developing settings: validation in a rural and urban Kenyan population. Reprod Health. 2017;14:1. doi: 10.1186/s12978-017-0381-7 28938885 PMC5610540

[19] Afulani PA, Altman MR, Castillo E, et al. Development of the person-centered prenatal care scale for people of color. Am J Obstet Gynecol. 2021;225:427. e1–427.e13. doi: 10.1016/j.ajog.2021.04.216 33862014

[20] Sheferaw ED, Mengesha TZ, Wase SB. Development of a tool to measure women’s perception of respectful maternity care in public health facilities. BMC Pregnancy Childbirth. 2016;16:1. doi: 10.1186/s12884-016-0848-5 27026164 PMC4810502

[21] Rubashkin N, Szebik I, Baji P, et al. Assessing quality of maternity care in Hungary: expert validation and testing of the mother-centered prenatal care (MCPC) survey instrument. Reprod Health. 2017;14:1–10. doi: 10.1186/s12978-017-0413-3 29145863 PMC5689156

[22] Limmer CM, Stoll K, Vedam S, et al. Measuring disrespect and abuse during childbirth in a high-resource country: development and validation of a German self-report tool. Midwifery. 2023;126:103809. doi: 10.1016/j.midw.2023.103809 37689053

[23] Vedam S, Stoll K, Rubashkin N, et al. The mothers on respect (MOR) index: measuring quality, safety, and human rights in childbirth. SSM Popul Health. 2017;3:201–210. doi: 10.1016/j.ssmph.2017.01.005 29349217 PMC5768993

[24] Dynes MM, Twentyman E, Kelly L, et al. Patient and provider determinants for receipt of three dimensions of respectful maternity care in Kigoma Region, Tanzania. Reprod Health. 2018;15:15. doi: 10.1186/s12978-018-0486-7 29506559 PMC5838967

[25] Dzomeku VM, Boamah Mensah AB, Nakua KE, et al. Developing a tool for measuring postpartum women’s experiences of respectful maternity care at a tertiary hospital in Kumasi, Ghana. Heliyon. 2020;6:1. doi: 10.1016/j.heliyon.2020.e04374 PMC7355990 32685719

[26] The United Republic of Tanzania Ministry of Health and Social Welfare. Annual health sector performance profile 2020. Dar es Salaam, Tanzania: Ministry of Health; 2021.

[27] Ministry of Health, Community Development, Gender E and C (MoHCDGEC) [Tanzania, Mainland], Ministry of Health (MoH) [Zanzibar], National Bureau of Statistics (NBS) O of the C, Government Statistician (OCGS) and ICF 2016. Tanzania demographic and health survey and malaria indicator survey (TDHS-MIS) 2015-16. Dar es Salaam, Tanzania: Ministry of Health; 2016.

[28] Ministry of Health (MoH) [Tanzania Mainland], Ministry of Health (MoH) [Zanzibar], National Bureau of Statistics (NBS), Office of the Chief Government Statistician (OCGS), ICF. Tanzania demographic and health survey and malaria indicator survey (TDHS-MIS), 2022. Dar es Salaam, Tanzania: Ministry of Health; 2023.

[29] The United Republic of Tanzania Ministry of Health and Social Welfare. National guideline for gender and respectful care mainstreaming and integration across RMNCAH services in Tanzania. Dar es Salaam, Tanzania: Ministry of Health; 2019.

[30] Sequeira Dmello B, Sellah Z, Magembe G, et al. Learning from changes concurrent with implementing a complex and dynamic intervention to improve urban maternal and perinatal health in dar es salaam. BMJ Glob Health. 2021;6:6e004022. doi: 10.1136/bmjgh-2020-004022 PMC7825259 33479018

[31] Necochea E, Tripathi V, Kim YM, et al. Implementation of the standards-based management and recognition approach to quality improvement in maternal, newborn, and child health programs in low-resource countries. Int J Gynecology Obstet. 2015;130:S17–24. doi: 10.1016/j.ijgo.2015.04.003 26115852

[32] Vargas C, Whelan J, Brimblecombe J, et al. Co-creation, co-design and co-production for public health: a perspective on definitions and distinctions. Public Health Res Pract. 2022;32:2. doi: 10.17061/phrp3222211 35702744

[33] Maaløe N, Housseine N, Sørensen JB, et al. Scaling up context-tailored clinical guidelines and training to improve childbirth care in urban, low-resource maternity units in Tanzania: a protocol for a stepped-wedged cluster randomized trial with embedded qualitative and economic analyses (the PartoMa scale-up study). Glob Health Action. 2022;15:2034135. 35410590 10.1080/16549716.2022.2034135 PMC9009913

[34] Fischer M, Safaeinili N, Haverfield MC, et al. Approach to human-centered, evidence-driven adaptive design (AHEAD) for health care interventions: a proposed framework. J Gen Intern Med. 2021;36:1041–1048. doi: 10.1007/s11606-020-06451-4 33537952 PMC8042058

[35] Office of the Chief Government Statistician. The United Republic of Tanzania national population projections. 2018.

[36] The United Republic of Tanzania Ministry of Health and Social Welfare. National health sector strategic plan (2021-2026). Dar es Salaam, Tanzania: Ministry of Health; 2021.

[37] Sequeira Dmello B, John TW, Housseine N, et al. Incidence and determinants of perinatal mortality in five urban hospitals in Dar es Salaam, Tanzania: a cohort study with an embedded case–control analysis. BMC Pregnancy Childbirth. 2024;24:24. doi: 10.1186/s12884-023-06096-1 38218766 PMC10787400

[38] Sando D, Ratcliffe H, McDonald K, et al. The prevalence of disrespect and abuse during facility-based childbirth in urban Tanzania. BMC Pregnancy Childbirth. 2016;16:1–10. doi: 10.1186/s12884-016-1019-4 27543002 PMC4992239

[39] Sando D, Abuya T, Asefa A, et al. Methods used in prevalence studies of disrespect and abuse during facility-based childbirth: lessons learned. Reprod Health. 2017;14:1–18. doi: 10.1186/s12978-017-0389-z 29020966 PMC5637332

[40] Sørensen JB, Housseine N, Maaløe N, et al. Scaling up locally adapted clinical practice guidelines for improving childbirth care in Tanzania: a protocol for programme theory and qualitative methods of the PartoMa scale-up study. Glob Health Action. 2022;15:15. doi: 10.1080/16549716.2022.2034136 PMC8942528 35311627

[41] Shove E, Pantzar M, Watson M. The dynamics of social practice: everyday life and how it changes. London: Sage Publications; 2012.

[42] Sekhon M, Cartwright M, Francis JJ. Acceptability of healthcare interventions: an overview of reviews and development of a theoretical framework. BMC Health Serv Res. 2017;17:1–13. doi: 10.1186/s12913-017-2031-8 28126032 PMC5267473

[43] Sekhon M, Cartwright M, Francis JJ. Development of a theory-informed questionnaire to assess the acceptability of healthcare interventions. BMC Health Serv Res. 2022;22:1–12. doi: 10.1186/s12913-022-07577-3 35232455 PMC8887649

[44] DeVellis RF. Scale development: theory and applications. 2nd ed. Thousand Oaks (CA), USA: Sage; 2003.

[45] Bohren MA, Mehrtash H, Fawole B, et al. How women are treated during facility-based childbirth in four countries: a cross-sectional study with labour observations and community-based surveys. Lancet. 2019;394:1750–1763. doi: 10.1016/S0140-6736(19)31992-0 31604660 PMC6853169

[46] Bohren MA, Vogel JP, Fawole B, et al. Methodological development of tools to measure how women are treated during facility-based childbirth in four countries: labor observation and community survey. BMC Med Res Methodol. 2018;18:18. doi: 10.1186/s12874-018-0603-x 30442102 PMC6238369

[47] Ramsey K, Moyo W, Larsen A, et al. Staha project: building understanding of how to promote respectful and attentive care in Tanzania. Dar es Salaam; 2015.

[48] World Health Organization. Standards for improving quality of maternal and newborn care in health facilities. Geneva: World Health Organization; 2016.

[49] Asefa A, Morgan A, Gebremedhin S, et al. Mitigating the mistreatment of childbearing women: evaluation of respectful maternity care intervention in Ethiopian hospitals. BMJ Open. 2020;10:1. doi: 10.1136/bmjopen-2020-038871 PMC7473661 32883738

[50] Mselle LT, Kohi TW, Dol J. Humanizing birth in Tanzania: a qualitative study on the (mis) treatment of women during childbirth from the perspective of mothers and fathers. BMC Pregnancy Childbirth. 2019;19:1–11. doi: 10.1186/s12884-019-2385-5 31277609 PMC6612108

[51] Das G, Masoi TJ, Kibusi SM, et al. Patient and provider perspectives of disrespect and abuse during childbirth in Tanzania: a literature review. Open J Obstet Gynecol. 2021;11:1248–1272. doi: 10.4236/ojog.2021.119118

[52] Scott K, Gharai D, Sharma M, et al. Yes, no, maybe so: the importance of cognitive interviewing to enhance structured surveys on respectful maternity care in northern India. Health Policy Plan. 2020;35:67–77. doi: 10.1093/heapol/czz141 31670773 PMC7053388

[53] Bishanga DR, Massenga J, Mwanamsangu AH, et al. Women’s experience of facility-based childbirth care and receipt of an early postnatal check for herself and her newborn in Northwestern Tanzania. Int J Environ Res Public Health. 2019;16:481. doi: 10.3390/ijerph16030481 30736396 PMC6388277

[54] Mengistie T, Mulatu T, Alemayehu A, et al. Respectful maternity care among women who gave birth at public hospitals in Hadiya Zone, Southern Ethiopia. Front Public Health. 2022;10:949943. doi: 10.3389/fpubh.2022.949943 36238243 PMC9551054

[55] Freedman LP, Kruk ME. Disrespect and abuse of women in childbirth: challenging the global quality and accountability agendas. The Lancet. 2014;384:e42–4. doi: 10.1016/S0140-6736(14)60859-X 24965825

[56] Sando D, Kendall T, Lyatuu G, et al. Disrespect and abuse during childbirth in Tanzania: are women living with HIV more vulnerable? J Acquir Immune Defic Syndr. 2014;67:S228–34. doi: 10.1097/QAI.0000000000000378 25436822 PMC4251905

[57] Benova L, Moller AB, Hill K, et al. What is meant by validity in maternal and newborn health measurement? A conceptual framework for understanding indicator validation. PLOS ONE. 2020;15:5. doi: 10.1371/journal.pone.0233969 PMC7259779 32470019

[58] Benova L, Moller AB, Moran AC, et al. “What gets measured better gets done better”: the landscape of validation of global maternal and newborn health indicators through key informant interviews. PLoS One. 2019;14:11. doi: 10.1371/journal.pone.0224746 PMC6830807 31689328

