# Supplementary material for: ‘I am happy to be listened to’: co-creation of a simple tool to measure women’s experiences of respectful maternity care in urban Tanzania
Source: Glob Health Action. 2024 Sep 24;17(1):2403972. doi: 10.1080/16549716.2024.2403972 (PMC11423523; doi:10.1080/16549716.2024.2403972)
Supplement: SupplementaryFile8_Tool_ENGLISH.docx [file ZGHA_A_2403972_SM6966.docx]

Facility Name/ID: Date of interview:

**Screening**

1. Which facility did you give birth in your recent pregnancy? ___________________

*If she did not deliver in facility 1-4, STOP the interview. Otherwise proceed*

1. How did you give birth in your recent pregnancy? (Pease tick one box)

- Normal vaginal delivery
- Vacuum delivery
- Planned caesarean section
- emergency caesarean section

*If she had a planned caesarean section (upasuaji uliopangwa), STOP the interview. Otherwise proceed*

**Information**

We are trying to understand women’s experiences of giving birth in this (dhospital in order to improve the care for them and their babies. We will ask you questions related to the services you received during your labor and delivery. There is no right or wrong answer. Nothing you tell us will be associated with your name, your child's name or the ability of you and your family to get care in the hospital in the future. Also, your answers will not affect your current care. It will take me ten minutes to do this exercise, do you have a question and are you ready?

- Verbal consent obtained

**General characteristics of women**

1. How old are you*? __________________*
2. What is your highest level of formal education? (Please tick one box)
   1. I have not attended school or have not completed primary school
   2. I have complete primary school
   3. I have completed secondary school (Form IV or VI)
   4. I have completed high level education (university level education)
3. What is your current marital status? (Please tick one box)
4. Single
5. Married
6. Cohabiting
7. Other
8. How many times have you given birth (include this pregnancy)? ____________________
9. On which date did you give birth in your recent pregnancy? ___________________
10. Around what time did you give birth in your recent pregnancy? ___________________
11. Which facility did you give birth in your recent pregnancy? ______________________
12. How did you give birth in your recent pregnancy? (Please tick one box)
13. Normal vaginal delivery
14. Vacuum delivery
15. Planned caesarean section
16. emergency caesarean section
17. Did you have any complication/problem during childbirth?
18. No
19. Yes
20. If yes, please specify the problem/complication you had during childbirth? _______________________________________________________________________
21. How is your baby doing? ________________________________________

**MISTREATMENT**

Please indicate with a √ mark your answer if it is **yes** or if it is **no**, or **I don't know** for the following questions. If there is an explanation, write it in the margin.

|  | | No | Yes | I don’t know | Explanation |
| --- | --- | --- | --- | --- | --- |
|  | Did the healthcare workers treat you kindly by for example speaking to you in a kind voice, smiling at you, making you feel welcomed and saying encouraging and comforting words to you during your pain? |  |  |  |  |
|  | Do you feel like the healthcare workers at the facility gave you a chance to ask questions and listened to your concerns and wishes? |  |  |  |  |
|  | Did the healthcare workers explain to you why they were doing examinations or procedures on you or giving you medication? |  |  |  |  |
|  | During your time in hospital did you receive any medication or procedures without permission/consent beforehand? Examples of procedures include vaginal examination, caesarean section, episiotomy, removal of the uterus, tubal ligation, post- partum IUCD, manual removal of placenta, assisted vaginal delivery. |  |  |  |  |
|  | Do you feel healthcare workers neglected or ignored you? |  |  |  |  |
|  | Do you feel you received poor care because of any of the following: your physical appearance, ethnicity, race, tribe, culture or religion, age, marital status, number of children you have, your education, wealth, inability to pay hospital bills, HIV status? |  |  |  |  |
|  | Were you allowed to have someone you know, a close family member to stay with you during childbirth such as your mother, a friend, sister, mother-in-law, or your partner? |  |  |  |  |
|  | Were you told that you could walk and encouraged to move around during labour? |  |  |  |  |
|  | Were you allowed to eat and drink when you wanted to or when you were thirsty/hungry? |  |  |  |  |
|  | | No | Yes | I don’t know | Explanation |
|  | Was pain medication given during and after painful procedures, for example during repair of the perineum and after caesarean section? |  |  |  |  |
|  | Did a healthcare worker talk to you rudely? For example, did anyone shout at you, scold you, insult you, frighten you that something bad would happen if you didn’t cooperate or blame you for something that happened to you or your baby during your time in hospital? |  |  |  |  |
|  | During your stay in the hospital or during childbirth did any staff or anyone physically hurt you for example by doing any of the following: kicking, slapping, pushing, hitting, pressing hard on your abdomen, pinching you with fingers or any sharp instrument or cover your mouth or physically tie you up? |  |  |  |  |
|  | Did any healthcare worker make a flirting comment, or use inappropriate sexual language or requested you to have sex with them? |  |  |  |  |
|  | Do you feel that the care providers talked to you about your information out loud or in a way that other mothers or other health workers not involved in your care could hear? |  |  |  |  |
|  | During examinations in the ward, were you always covered up with a cloth or curtain so that you did not feel exposed? |  |  |  |  |
|  | At any time, did you have to share a bed /or mattress with another woman or women? |  |  |  |  |
|  | Immediately after birth, were you instructed to clean up your own blood, urine, faeces or birth ﬂuid from the birthing area/bed? |  |  |  |  |
|  | Did healthcare providers suggest or ask you (or your family or friends) for a bribe, informal payment, or gift? |  |  |  |  |
|  | Did you receive information about the condition of your baby during labour and after birth? |  |  |  |  |
|  | Was your baby separated from you after birth without information/explanation? |  |  |  |  |
|  | Were you or your baby denied treatment due to inability to pay? |  |  |  |  |
|  | Do you think there was enough health staff in the facility to care for you? |  |  |  |  |
|  | Do you think the facility had a clean environment and good toilet and washing facilities? |  |  |  |  |
|  | Do you think there were enough medicines and supplies in the facility to care for you? |  |  |  |  |
|  | Would you recommend this facility to other women for giving birth? |  |  |  |  |

1. Please mention ways to improve the supportive care/ respectful care that you received at this facility.

………………………………………………………………………………………………………………………………..

………………………………………………………………………………………………………………………………..

………………………………………………………………………………………………………………………………..

1. Are you satisfied (Do you have anything else you wish to say/ do you have any comments or questions for me?

………………………………………………………………………………………………………………………………..

………………………………………………………………………………………………………………………………..

………………………………………………………………………………………………………………………………..

1. Were the questions asked understandable? How did you feel when you answered the questions? Can you tell us how we can improve these questions?

………………………………………………………………………………………………………………………………..

………………………………………………………………………………………………………………………………..

………………………………………………………………………………………………………………………………..
