# Supplementary material for: ‘I am happy to be listened to’: co-creation of a simple tool to measure women’s experiences of respectful maternity care in urban Tanzania
Source: Glob Health Action. 2024 Sep 24;17(1):2403972. doi: 10.1080/16549716.2024.2403972 (PMC11423523; doi:10.1080/16549716.2024.2403972)
Supplement: SupplementaryFiles1_6.docx [file ZGHA_A_2403972_SM6965.docx]

**Supplementary File 1: Structured observations of the study health facilities’ contextual factors (e.g., human resources profiles, supplies constraints, and physical environment**

The study was conducted in four urban public hospitals that are CCBRT partner facilities and included in the PartoMa study. Three of these are regional referral hospitals and one is a primary maternity hospital, all providing 24-hour comprehensive obstetric care. Each handle 6,000 to 10,000 births annually, serving a diverse patient population including low-risk and referred cases from peripheral facilities. These resource-constrained hospitals, typical of low-income countries, primarily care for women from lower socioeconomic backgrounds. Birth attendants often manage 3-6 women simultaneously, with space constraints leading to shared beds and minimal privacy, although screens and curtains are used during examinations.

These facilities are involved in a regionally led quality improvement intervention, which includes training, infrastructure upgrades, and data quality enhancements. Despite these improvements, including a 41% reduction in births at congested hospitals and a six-fold increase in care quality, challenges such as stagnation in maternal mortality reduction and unchanged neonatal mortality persist. The intervention measures quality of intrapartum care using the Standards Based Management and Recognition (SBM-R) approach. The SBM-R tool addresses some aspects of interpersonal communication, supportive care, use of local language and birth position choice, however, it lacks standards for measuring mistreatment during birth.

Maternity care is subsidized by the government; however, patients are sometimes expected to ‘cost-share’ for medicines and investigations. Payment exemptions are managed by the social welfare department. Unofficial payments and bribes prohibited by policy.

All facilities display client charters and leadership contacts. They encourage patient suggestions through public announcements or through confidential feedback through suggestions boxes placed at the reception.

Birth companionship is not practiced. Relatives escort women and visit to bring food. They usually wait outside. They are not allowed to be present at the time of birth. For special cases, if extra help is needed, the midwives may allow a female relative to enter the wards, after birth and provide extra assistance.

**Supplementary File 2: A guide for FGDs/interviews to be conducted with birth attendants and managers to assess acceptability**

**Presentation of interview to the participant**

- I am a researcher and part of a Tanzanian and international team that looks into health care workers’ and managers’ experiences with working at the maternity wards at several hospitals in Dar es Salaam Region
- Thank you for taking the time to talk to me about respectful maternity care.
- With your permission, I would like you, as a group, to discuss the tool that we have collaboratively developed to measure respectful maternity care at maternity units in Dar es Salaam.
- You have been asked to take part in this FGD, because you were identified as an experienced birth attendant/manager in this hospital who can inform us about the acceptability and appropriateness of the developed tool for measuring mistreatment/disrespect of women around the time of birth. The aim is to improve respectful care.
- Your responses and perspectives will be kept strictly confidential. Your name will not appear anywhere. If you are uncomfortable participating in this FGD, you are free to let me know and we will stop the interview. If you want to take a break and continue at another time, please let me know.
- Your experiences are very important to us. Keep in mind that we are interested in both negative and positive comments. We would like to learn from your sincere opinions and experiences, so please do not fear to give your honest answers.
- There are no right or wrong things to say – we are only interested in your perspectives and recommendations.
- The interview will take approximately one hour and 30 minutes. Can I have your permission to participate in this focus group discussion?
- Do you have any questions before we start the focus group discussion?

| FOCUS GROUP AND TRANSCRIPT DATA | |
| --- | --- |
| Transcript code |  |
| Type of FGD |  |
| Date of FGD |  |
| Place and time of FGD start |  |
| FGD length |  |
| Name of facilitator |  |
| Name of transcriber/translator |  |
| Name of reviewer |  |

**Details of participants**

| **Name** | **Profession** | **Age** | **Years of experience with childbirth care** |
| --- | --- | --- | --- |
|  |  |  |  |
|  |  |  |  |
|  |  |  |  |
|  |  |  |  |
|  |  |  |  |
|  |  |  |  |
|  |  |  |  |
|  |  |  |  |

| **Questions** | **What do we want to know** |
| --- | --- |
| **Topic 1: Knowledge and perception about (dis)respectful maternity care.** | |
| 1. What is the meaning of RMC?   *-How should women be treated during childbirth?*  *-What are the different types of disrespect and abuse?*   1. Do you think that disrespectful care is a problem in your maternity unit? *Can you give some examples that you have seen at your hospital?* 2. What contributes to the problem of mistreatment/disrespect of women in your facility 3. How will this tool help you address this problem? -*-How will it affect the way you take care of women?*   *-Do you think it can help improve care?* | Awareness and knowledge of the problem  How will the tool help address this problem? |
| **Topic 2: Content of the tool** | |
| 1. What do you think about the topics covered in the tool?   -Should there be more or less?   1. What do you think about the length of the tool?   -*is too long or too short?*  *-Are there any questions that should be removed?* | To assess face validity of the tool |
| **Topic 3: Language and format of the tool** | |
| 1. Is the Swahili language of the tool understandable?   *-Is there any question, word or term that should be changed?*   1. What do you think of the layout of the tool?    - *E.g. The order of the questions.*    - *The font and size* | To assess whether the Swahili language used is appropriate and understandable.  To improve the presentation of the tool |
| **Topic 4**: **Administration of the tool** | |
| 1. How frequently do you think the tool should be administered? 2. Who should administer the tool?   -*e.g. staff versus self-administered, care of staff*   1. When should the be administered *e.g. before discharge from hospital, after discharge? And at what particular time?* 2. Describe to me the barriers that may hinder implementation of this intervention (individual, health systems)  - *What would make it easy/difficult for you to participate?* - *What problem do you anticipate with the implementation of this tool?* | To determine how the tool should be administered |
| **Topic 5: Other Suggestions** | |
| 1. What, if anything, would you like to change about the tool?  - *Do you have any other suggestions of how to improve the tool* |  |
| Closing: Thank you for your participation | |

| **Supplementary File 3: Scoping review of physical and verbal prevalence from cross-sectional studies (adapted from Sando et al., 2017)** | | | | | |
| --- | --- | --- | --- | --- | --- |
| Author | Study population | Sample size | Data collection methods | Physical Abuse | Verbal Abuse |
| Okafor et al | Women attending immunization clinics. Nigeria | 446 women | Exit interviews | 35.7%% | 29.6% |
| Kruk et al | Post-natal women, Tanzania | 1779 women | Exit interviews and community interviews | 2.90% | 12.89% |
| Abuya et al | Post-natal women. Kenya | 641 women | Exit interviews | 4.20% | 18% |
| Sando et al | Post-natal women. Tanzania | 1914 women | Exit interviews and community interviews | 5% | 6% |
| Asefa et al | Post-natal women who delivered SVD. Ethiopia | 173 women | Exit interviews | 32.90% | 12.10% |
| Hajizadeh et al | Post delivery women, Tabriz, Iran | 334 women | Exit interviews | 36.50% | 82.30% |
| Ukke et al | Post delivery women, Ethiopia | 281 women | Exit interviews | 29.50% | 36.70% |
| Azhar et al*** | Household survey of post-delivery women. Pakistan | 360 women | Household survey | 0.60% | 12.20% |
| Sethi et al | Women in labor and delivery. Malawi | 2109 women | Direct observation of labor and delivery process | 0.20% |  |

**Supplementary File 4**: **Mapping* of Respectful Maternity Care or Experience of Care measurement tools**

- Sando et al. The prevalence of disrespect and abuse during facility-based childbirth in urban Tanzania. BMC Pregnancy and Childbirth (2016) 16:236.
- Bohren MA et al. How women are treated during facility-based childbirth in four countries: a cross-sectional study with labour observations and community-based surveys. Lancet 2019; 394: 1750–63
- Bohren et al. Methodological development of tools to measure how women are treated during facility-based childbirth in four countries: labor observation and community survey. BMC Medical Research Methodology (2018) 18:132
- Afulani P et al. Development of a tool to measure person-centered maternity care in developing settings: Validation in a rural and urban Kenyan population, Reproductive Health (2017) 14(1)
- Sheferaw et al. Development of a tool to measure women's perception of respectful maternity care in public health facilities. BMC Pregnancy and Childbirth (2016) 16(1)

* This pragmatic mapping exercise, conducted in early 2020, aimed to inform the development of the zero draft, drawing extensively from the Comprehensive Community Survey Tool (Bohren et al., 2018).

| **Supplementary File 5: Cronbach Alpha on Sample of 838 with Final RMC-Tool** | |
| --- | --- |
| Item | alpha |
| Kindness | 0.5424 |
| Given chance to speak, ask questions/listened to you | 0.5420 |
| Given information on procedures/examination/treatment | 0.5465 |
| Given meds/procedures without your consent | 0.5448 |
| Neglected/abandoned you | 0.5376 |
| Discriminated/stigmatized you because of any particular characteristics | 0.5504 |
| Birth companion allowed? | 0.5738 |
| Encouraged to move around? | 0.5553 |
| Allowed to eat and drink? | 0.5510 |
| Given pain relief during cutting or after CS | 0.6115 |
| Spoken to rudely/shouted/verbal abuse/blamed you/threaten | 0.5348 |
| Physical abuse | 0.5612 |
| Sexual abuse | 0.5641 |
| Exposed confidential information | 0.5477 |
| Were you covered with drape/curtains | 0.5503 |
| Did you share beds | 0.5972 |
| Were you asked to clean up bed | 0.5696 |
| Asked for bribe or gift | 0.5649 |
| Given information about baby | 0.5628 |
| Separated from baby without explanation | 0.5640 |
| Denied treatment for you or baby due to lack of funds | 0.5647 |
| Do you think there were enough staff? | 0.5573 |
| Was hospital environment and toilets clean? | 0.5642 |
| Was there enough meds and equipment? | 0.5726 |
| Would you recommend this hospital to other women for childbirth? | 0.5575 |
| **Test scale** | **0.5699** |

| **Supplementary File 6: Comparison of RMC items across four tools** | | | | |  |  |
| --- | --- | --- | --- | --- | --- | --- |
| RMC items | RMC_T | Bohren* et al.,  2018 | Afulani et al., 2017 | Sheferaw  et al.,  2016 | Asefa*  et al.,  2020 |  |
| Physical abuse, | ✓ | ✓ | ✓ | ✓ | ✓ |  |
| Verbal abuse | ✓ | ✓ | ✓ | ✓ | ✓ |  |
| Unconsented procedures | ✓ | ✓ | ✓ | 🗶 | ✓ |  |
| Given information on care | ✓ | ✓ | ✓ | 🗶 | ✓ |  |
| Privacy (information) | ✓ | ✓ | ✓ | 🗶 | ✓ |  |
| Privacy (physical) | ✓ | ✓ | ✓ | 🗶 | ✓ |  |
| Pain relief | ✓ | ✓ | ✓ | ✓ | 🗶 |  |
| Neglect/delay in care | ✓ | ✓ | ✓ | ✓ | ✓ |  |
| Listening/able to ask questions | ✓ | ✓ | ✓ | 🗶 | 🗶 |  |
| Labor support/ Birth companion | ✓ | ✓ | ✓ | 🗶 | ✓ |  |
| Clean hospital | ✓ | ✓ | ✓ | 🗶 | 🗶 |  |
| Enough staff | ✓ | ✓ | ✓ | 🗶 | 🗶 |  |
| Sexual abuse | ✓ | ✓ | 🗶 | 🗶 | 🗶 |  |
| Stigma/discrimination | ✓ | ✓ | 🗶 | ✓ | ✓ |  |
| Encouraged to eat and drink | ✓ | ✓ | 🗶 | 🗶 | ✓ |  |
| Encouraged to move | ✓ | ✓ | 🗶 | 🗶 | ✓ |  |
| Enough medicines /supplies | ✓ | ✓ | 🗶 | 🗶 | 🗶 |  |
| Asked for informal payments | ✓ | ✓ | 🗶 | 🗶 | 🗶 |  |
| Made to clean up after birth | ✓ | ✓ | 🗶 | 🗶 | 🗶 |  |
| Use local Language | 🗶 | ✓ | ✓ | ✓ | ✓ |  |
| Allowed preferred birth position | 🗶 | ✓ | ✓ | 🗶 | ✓ |  |
| Client involvement in care/not passive | 🗶 | ✓ | ✓ | ✓ | 🗶 |  |
| Kindness during care | ✓ | 🗶 | 🗶 | ✓ | 🗶 |  |
| Friendly | 🗶 | 🗶 | ✓ | ✓ | 🗶 |  |
| Treated with respect | 🗶 | ✓ | ✓ | ✓ | 🗶 |  |
| Called client by name, | 🗶 | 🗶 | ✓ | ✓ | 🗶 |  |
| Safe traditional/cultural practices | 🗶 | ✓ | 🗶 | ✓ | ✓ |  |
| Satisfaction, shared beds, denied treatment due to lack of ability to pay, mother separated from baby without consent, given information on baby | ✓ | ✓ | 🗶 | 🗶 | 🗶 |  |
| Painful vaginal exams, objectiﬁcation of women, poor staff attitudes, detainment in facilities, unclear fee structures, lack of redress | 🗶 | ✓ | 🗶 | 🗶 | 🗶 |  |
| Felt safe, trust, took best care, staff introduced self  support anxiety, talk about feeling, access to clean water, electricity, crowded | 🗶 | 🗶 | ✓ | 🗶 | 🗶 |  |
| Empathetic, attentive, delayed services due to health facility internal problem | 🗶 | ✓ | 🗶 | ✓ | 🗶 |  |
| *Adapted for ease of comparison—only main domains included (sub-scales excluded). | | | | | | |
|  | | | | |  |  |
